# Supplementary material for: Spatial and temporal dynamics of West Nile virus between Africa and Europe
Source: Nat Commun. 2023 Oct 13;14:6440. doi: 10.1038/s41467-023-42185-7 (PMC10575862; doi:10.1038/s41467-023-42185-7)
Supplement: Supplementary file 1 — Supplementary Information [file 41467_2023_42185_MOESM1_ESM.pdf]

Spatial and temporal dynamics of West Nile virus between Africa and Europe

Mencattelli[†], Ndione[†], Silverj[†] *et al.*

Supplementary information

Supplementary Fig. 1 - Maximum likelihood phylogeny of 228 WNV L1 genomes.

Supplementary Fig. 2 - Maximum likelihood phylogeny of 297 WNV L2 genomes.

Supplementary Fig. 3 - Downsampling of the WNV L1 dataset.

Supplementary Fig. 4 - Downsampling of the WNV L2 dataset.

Supplementary Fig. 5 - Root-to-tip divergence analysis WNV L1.

Supplementary Fig. 6 - Root-to-tip divergence analysis WNV L2.

Supplementary Fig. 7 - Sensitivity analysis of WNV L1 and L2 datasets - effect on ancestral node locations.

Supplementary Fig. 8 - Sensitivity analysis for testing the robustness of the phylogeographic inference of WNV L1.

Supplementary Fig. 9 - Sensitivity analysis for testing the robustness of the phylogeographic inference of WNV L2.

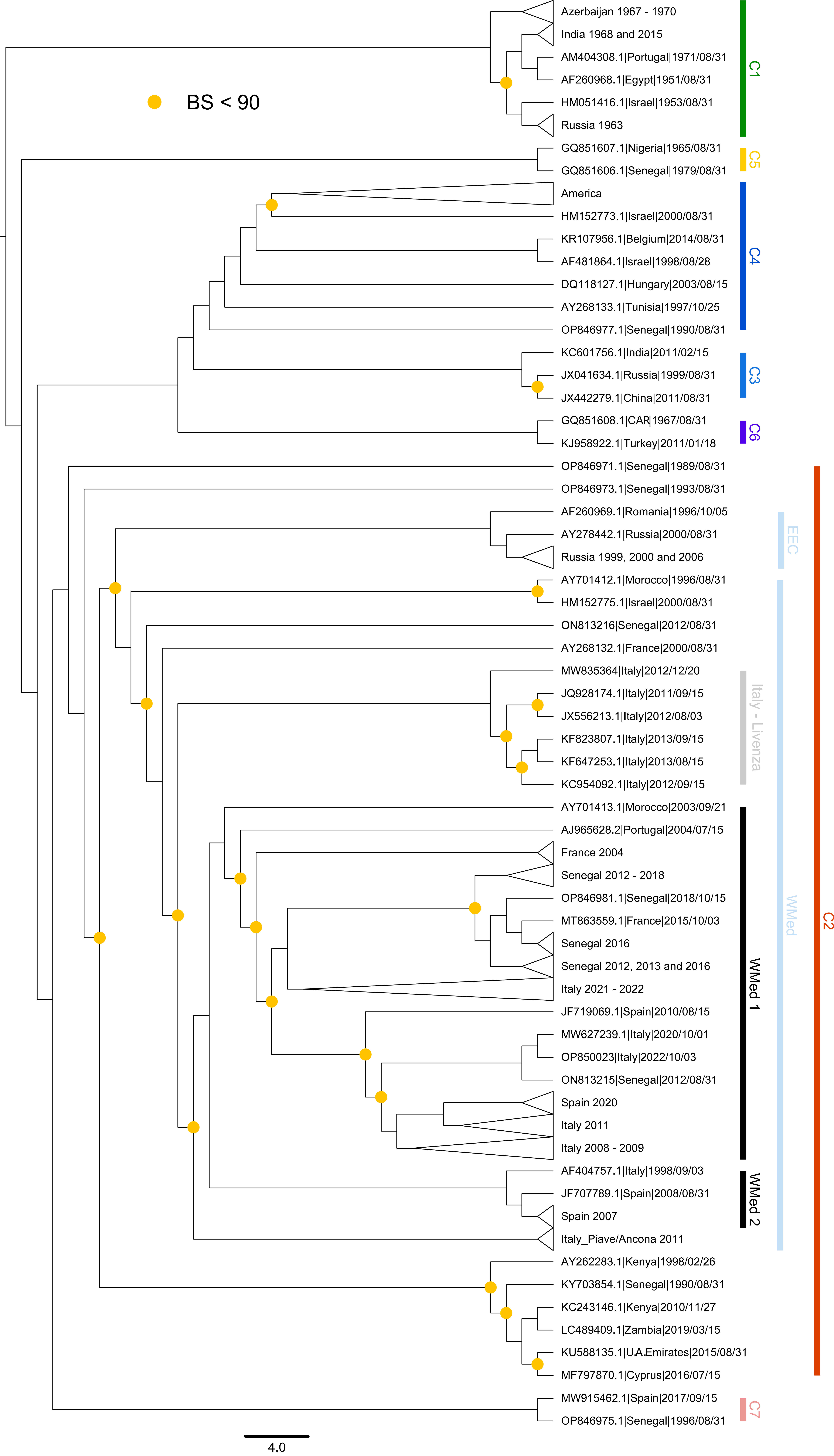

Supplementary Fig. 1. Maximum likelihood phylogeny of 228 WNV L1 genomes. Nodes with bootstrap supports (BS) lower than 90 are depicted with light orange dots. A scale bar at the bottom of the figure indicates the number of substitutions per site. All samples belong to clade 1, which is divided into different clusters (C1-C7) highlighted with colour bars on the right. Other relevant groups mentioned in the discussion are annotated in the same way: Eastern-European clade (EEC), Western Mediterranean clade (WMed, which is divided into 2 subclades, WMed1 and WMed2) and the Italian clade sampled near Livenza in 2011-2013.

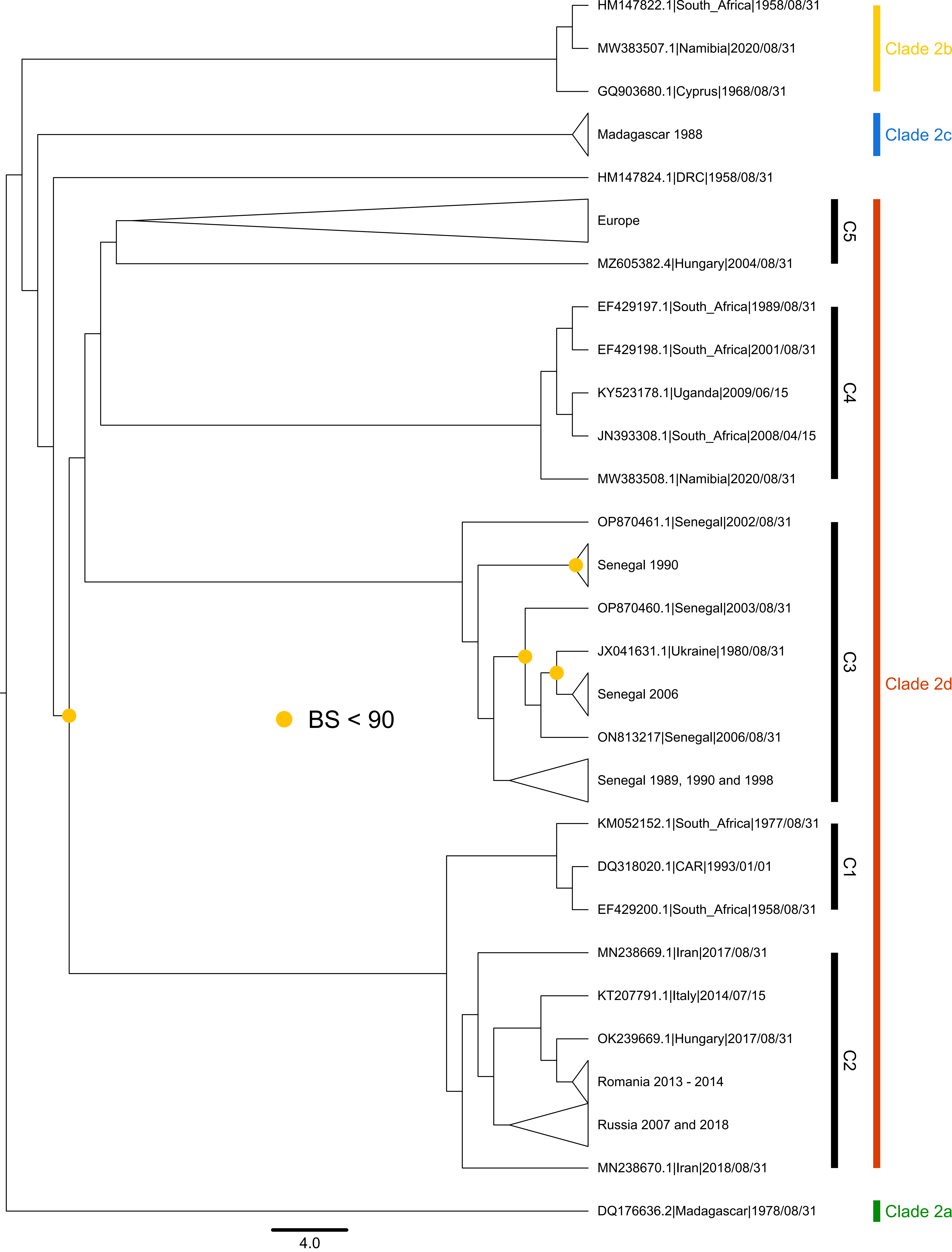

Supplementary Fig. 2. Maximum likelihood phylogeny of 297 WNV L2 genomes. Nodes with bootstrap supports (BS) lower than 90 are depicted with light orange dots. A scale bar at the bottom of the figure indicates the number of substitutions per site. Clades (2a-d) are indicated by coloured bars on the right side of the figure. Clade 2d is further divided into 5 clusters, annotated with black bars on the right side of the figure.

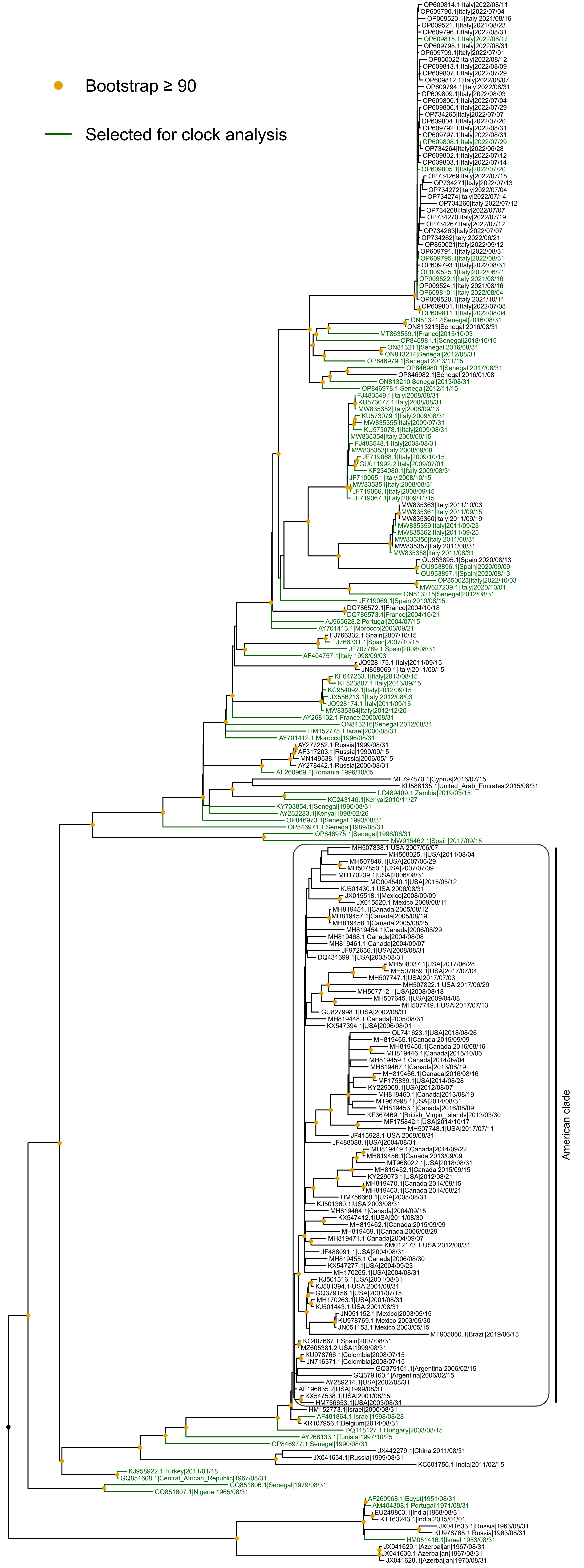

Supplementary Fig. 3. Downsampling of the WNV L1 dataset. Midpoint-rooted tree for the maximum likelihood analysis of 228 WNV L1 genomes is shown. Sequences that were selected for phylogeographic analysis are coloured in green. Nodes with bootstrap supports (BS) ≥ 90 are depicted with light orange dots. Nodes with no bootstrap are indicated with a black dot.

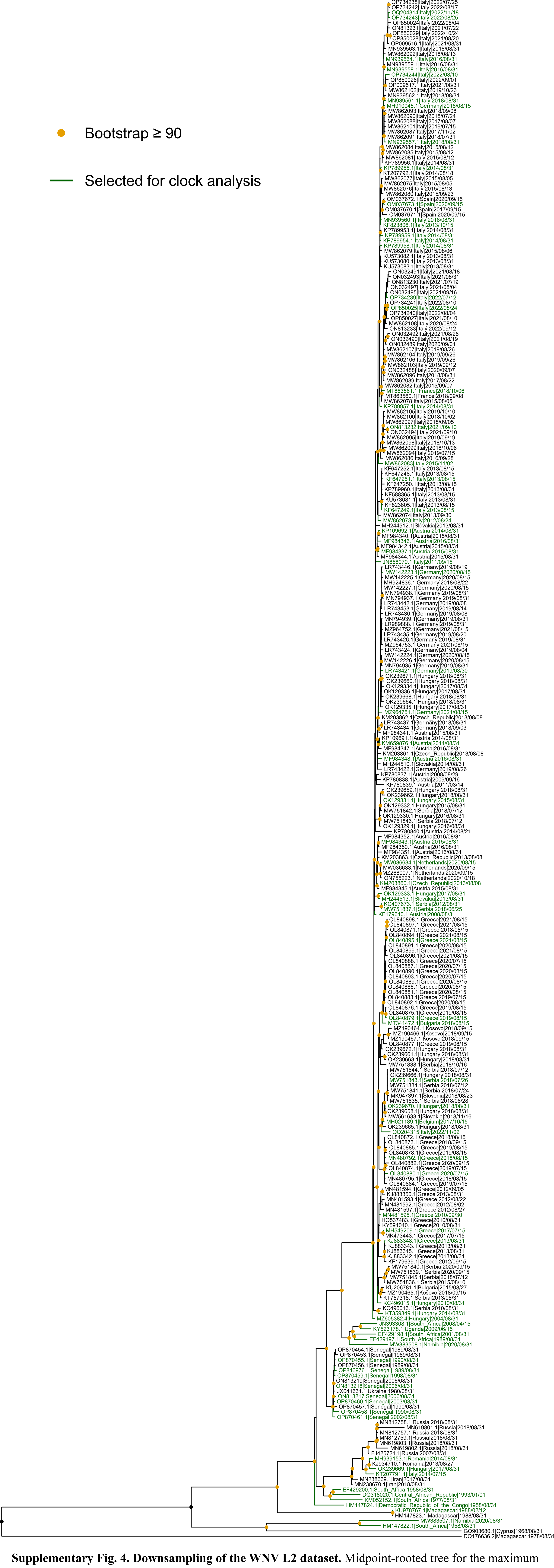

Supplementary Fig. 4. Downsampling of the WNV L2 dataset. Midpoint-rooted tree for the maximum likelihood analysis of 297 WNV L2 genomes is shown. Sequences that were selected for phylogeographic analysis are coloured in green. Nodes with bootstrap supports (BS) ≥ 90 are depicted with light orange dots. Nodes with no bootstrap are indicated with a black dot.

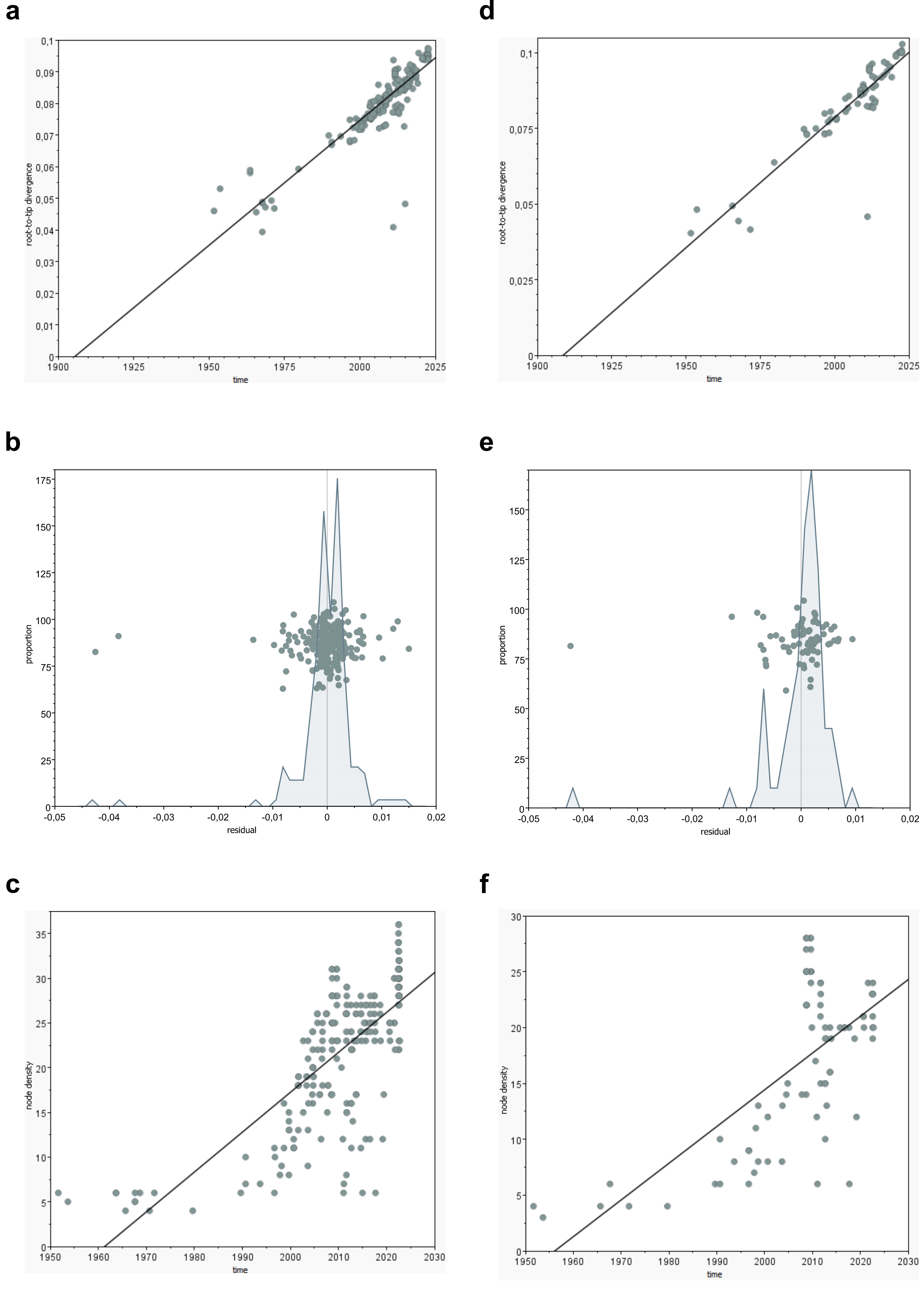

Supplementary Fig. 5. Root-to-tip divergence analysis WNV L1. A summary of the root-to-tip divergence analysis for the WNV L1 full dataset used for the maximum likelihood analysis (a, b, c) and for the reduced one used for Bayesian phylogeographic inference (d, e, f) is shown in the figure. a-d) Root-to-tip regression line showing a positive correlation between root-to-tip divergence and time. b-e) Residuals of the regression analysis. c-f) Positive correlation between node density and time.

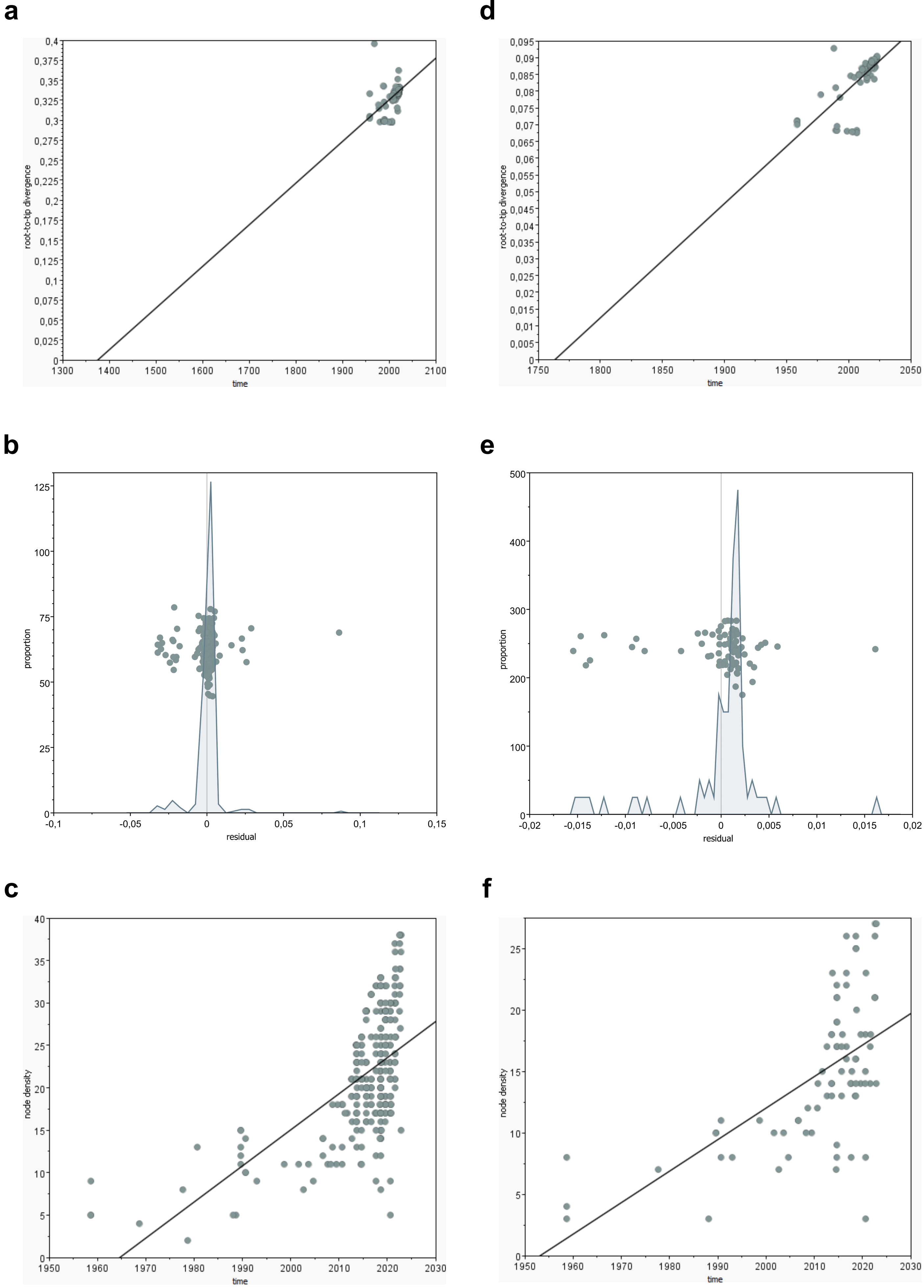

Supplementary Fig. 6. Root-to-tip divergence analysis WNV L2. A summary of the root-to-tip divergence analysis for the WNV L2 full dataset used for the maximum likelihood analysis (a, b, c) and for the reduced one used for Bayesian phylogeographic inference (d, e, f) is shown in the figure. a-d) Root-to-tip regression line showing a positive correlation between root-to-tip divergence and time. b-e) Residuals of the regression analysis. c-f) Positive correlation between node density and time.

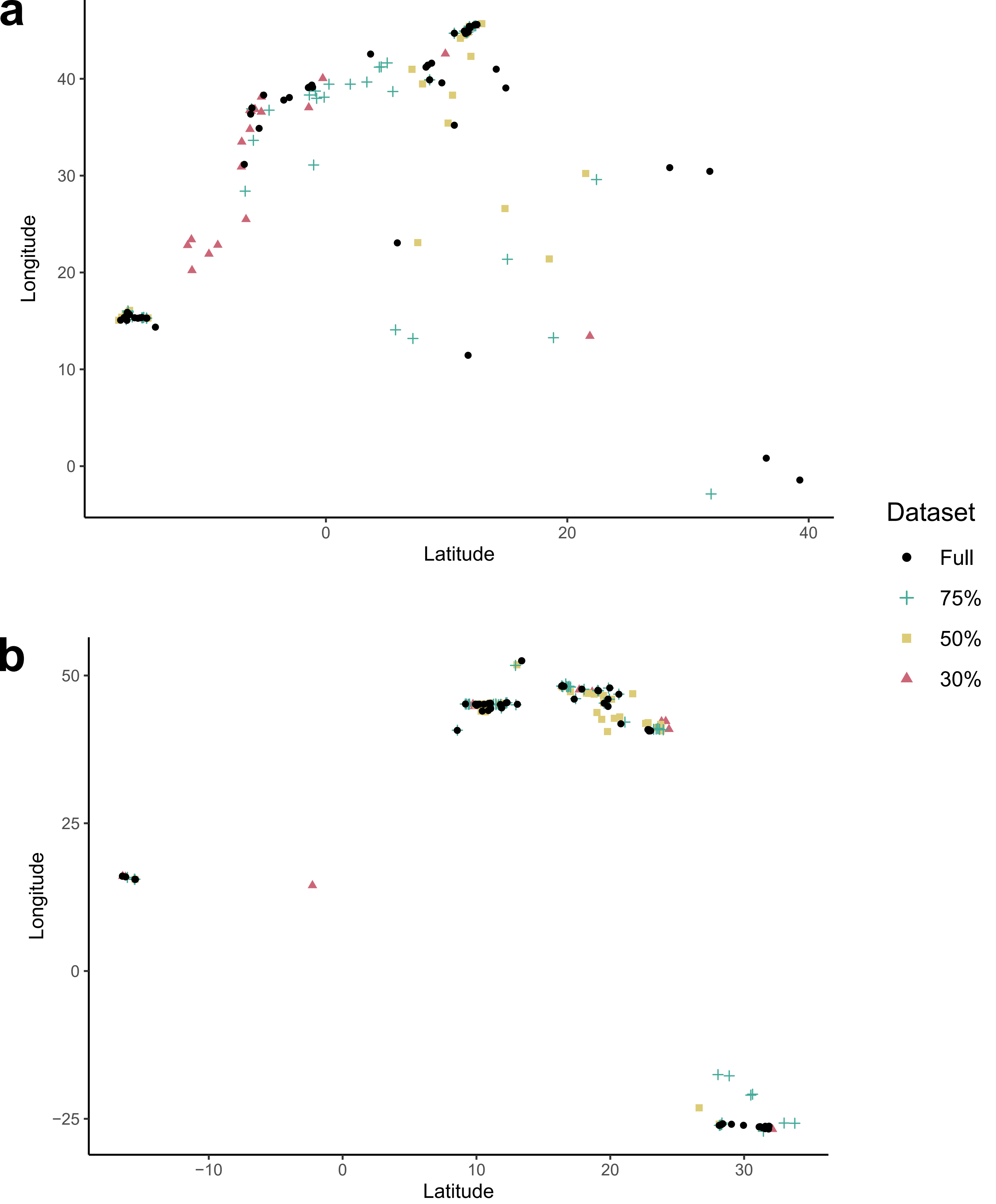

Supplementary Fig. 7. Sensitivity analysis of WNV L1 and L2 datasets - effect on ancestral node locations. Scatterplot showing the coordinates (latitude and longitude expressed in decimal degrees) of the inferred ancestral node locations, for the full dataset and for its different subsets sampled in the sensitivity analysis (different retained subsets of the main dataset are indicated by colour and shape: 100%: black dot; 75%: green cross; 50%: yellow square; 20%: light-red triangle). Data for WNV L1 a) and WNV L2 b) tend to overlap, with small differences among all datasets.

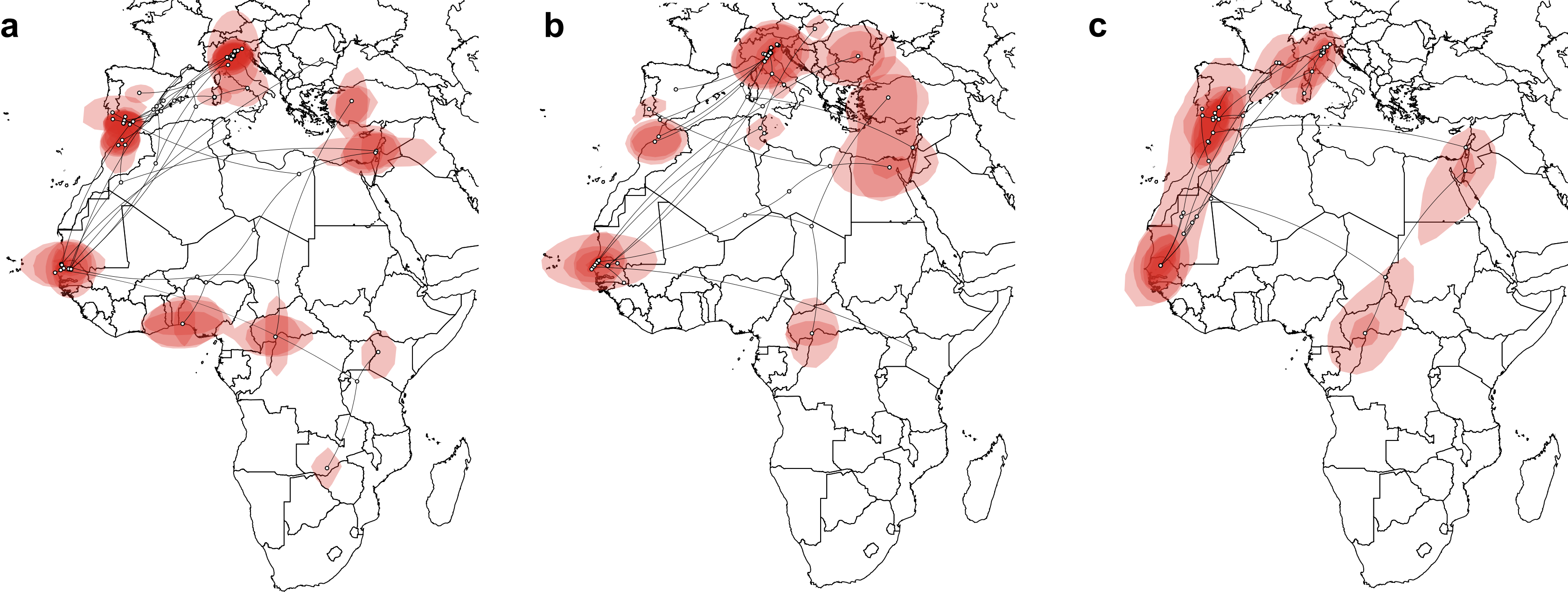

Supplementary Fig. 8. Sensitivity analysis for testing the robustness of the phylogeographic inference of WNV L1. Phylogeographic reconstructions for three subsets of the main dataset (a) 75%, b) 50% and c) 30%) returned very similar scenarios, always showing the presence of a corridor that virtually connects Senegal, Morocco, and southern Europe. Red shadows indicate the 80%HPDs of the inferred location, coloured by the inferred median date for that area, with darker hue indicating older areas of circulation.

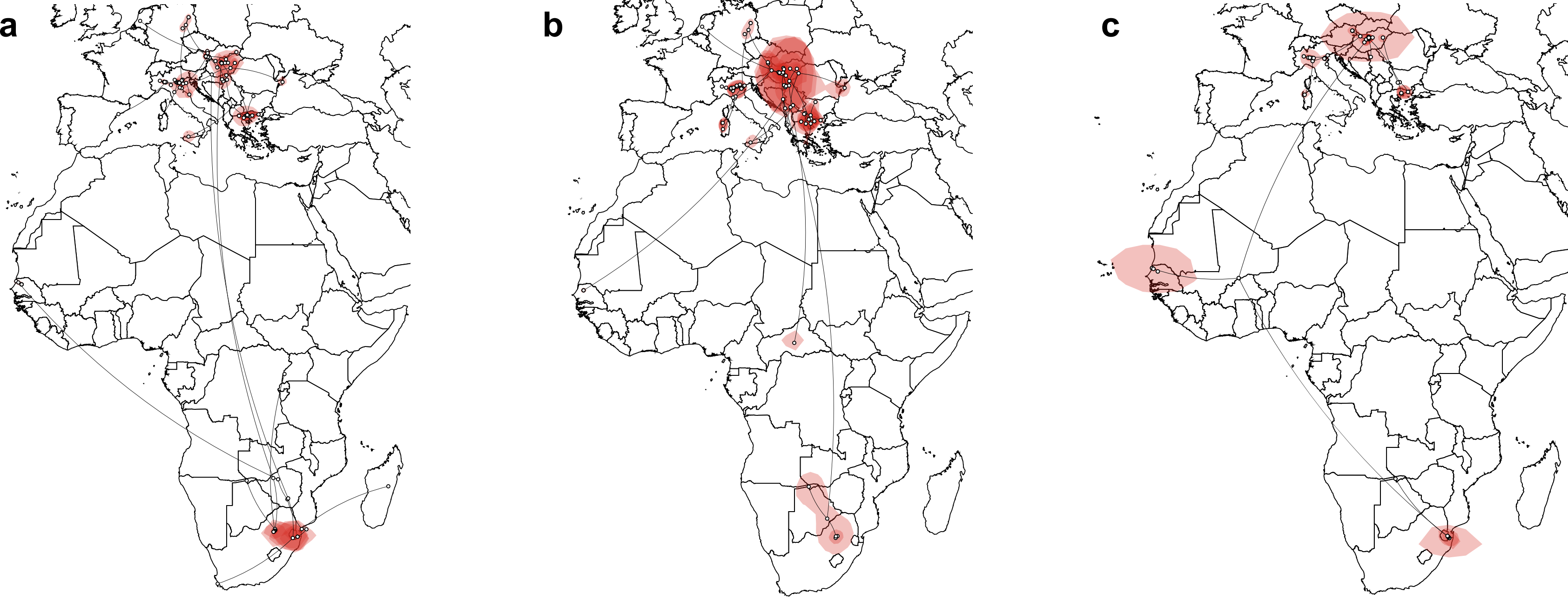

Supplementary Fig. 9. Sensitivity analysis for testing the robustness of the phylogeographic inference of WNV L2. Phylogeographic reconstructions for three subsets of the main dataset (a) 75%, b) 50% and c) 30%) returned very similar scenarios, with a major introductory event from southern Africa to central-eastern Europe (with the exception of b), in which the direction is reversed, as the random sampling step included only very recent African sequences, without old genomes). Red shadows indicate the 80%HPDs of the inferred location, coloured by the inferred median date for that area, with darker hue indicating older areas of circulation.
