# Supplementary material for: Spatial and temporal dynamics of West Nile virus between Africa and Europe
Source: Nat Commun. 2023 Oct 13;14:6440. doi: 10.1038/s41467-023-42185-7 (PMC10575862; doi:10.1038/s41467-023-42185-7)
Supplement: Supplementary file 3 — Description of additional supplementary files [file 41467_2023_42185_MOESM3_ESM.pdf]

**Description of additional supplementary files**

Supplementary Data 1.

Title: West Nile virus genome sequences curated metadata.

Description: A table of curated metadata of West Nile virus lineages 1 and 2 obtained at IZS-Teramo and IPD-Dakar and used in the paper.

Supplementary Data 2.

Title: West Nile virus genome sequences downloaded from NCBI.

Description: A table of curated metadata of West Nile virus lineages 1 and 2 genome sequences obtained from NCBI and used in the paper.

Supplementary Data 3.

Title: Geographic coordinates of West Nile virus sequences used for molecular clocks.

Description: A table of geographic coordinates of West Nile virus lineages 1 and 2 sequences used for molecular clock analyses.

Supplementary Data 4.

Title: Complete results for recombinant sequences.

Description: Results of the RDP4 analysis, showing all output parameters for detected recombinants.

Supplementary Data 5.

Title: Root-to-tip divergence analysis table.

Description: Table showing the full results of the root-to-tip regression analyses of all WNV L1 and WNV L2 datasets analysed in the paper.

Supplementary Data 6.

Title: Model selection results.

Description: Table showing the log Bayes factors obtained from subtracting to the -log values of the estimated marginal likelihood of the most favourite model (relaxed clock with a Bayesian Skyline tree prior) the same estimated values for all the other models.
