# Supplementary material for: Spatial and temporal dynamics of West Nile virus between Africa and Europe
Source: Nat Commun. 2023 Oct 13;14:6440. doi: 10.1038/s41467-023-42185-7 (PMC10575862; doi:10.1038/s41467-023-42185-7)
Supplement: Supplementary file 10 — Reporting Summary [file 41467_2023_42185_MOESM10_ESM.pdf]

nature portfolio

Corresponding author(s): Giulia Mencattelli

Last updated by author(s): Aug 29, 2023

Reporting Summary

Nature Portfolio wishes to improve the reproducibility of the work that we publish. This form provides structure for consistency and transparency in reporting. For further information on Nature Portfolio policies, see our Editorial Policies and the Editorial Policy Checklist.

Statistics

For all statistical analyses, confirm that the following items are present in the figure legend, table legend, main text, or Methods section.

n/a	Confirmed	
x		The exact sample size (*n*) for each experimental group/condition, given as a discrete number and unit of measurement
x		A statement on whether measurements were taken from distinct samples or whether the same sample was measured repeatedly
	x	The statistical test(s) used AND whether they are one- or two-sided<br>*Only common tests should be described solely by name; describe more complex techniques in the Methods section.*
x		A description of all covariates tested
x		A description of any assumptions or corrections, such as tests of normality and adjustment for multiple comparisons
	x	A full description of the statistical parameters including central tendency (e.g. means) or other basic estimates (e.g. regression coefficient) AND variation (e.g. standard deviation) or associated estimates of uncertainty (e.g. confidence intervals)
	x	For null hypothesis testing, the test statistic (e.g. *F*, *t*, *r*) with confidence intervals, effect sizes, degrees of freedom and *P* value noted<br>*Give P values as exact values whenever suitable.*
	x	For Bayesian analysis, information on the choice of priors and Markov chain Monte Carlo settings
x		For hierarchical and complex designs, identification of the appropriate level for tests and full reporting of outcomes
x		Estimates of effect sizes (e.g. Cohen's *d*, Pearson's *r*), indicating how they were calculated

Our web collection on statistics for biologists contains articles on many of the points above.

Software and code

Policy information about availability of computer code

Data collection	Using a custom R script for automated sequence retrieval, a new search was performed on the NCBI on 04/01/23, and 37 newly published worldwide WNV L1 and 39 WNV L2 sequences >= 200 nt were downloaded. The code to retrieve and filter sequences is available at https://github.com/andrea-silverj/WNV-Afr_Eur/tree/main/scripts (DOI: https://zenodo.org/badge/latestdoi/611814462)

Data analysis	The code used in the analyses is available at https://github.com/andrea-silverj/WNV-Afr_Eur/tree/main/scripts (DOI: https://zenodo.org/badge/latestdoi/611814462).<br>Assembly was automatically performed at the end of the sequencing run using the 'National Reference Centre for Whole Genome Sequencing of microbial pathogens: database and bioinformatic analysis' (GENPAT) platform formally established at the 'Istituto Zooprofilattico Sperimentale dell'Abruzzo e del Molise' (IZSAM) in Teramo (ltaly) as described in "Di Pasquale, A. et al. SARS-CoV-2 Surveillance in ltaly through Phylogenomic lnferences Based on Hamming Distances Derived from Pan-SNPs, -MNPs and -lnDels. BMC Genom. 2021, 22, 1-14". Consensus sequences were obtained using iVar v 1.3.1 after mapping trimmed reads to the WNV L2 MN652880 (Greece, 2018) and WNV L1 FJ483548 (ltaly, 2008) reference sequences, by using Snippy (https://github.com/tseemann/snippy). Sequence alignment was conducted using MAFFTv7.490 (https://mafft.cbrc.jp/alignment/server/) and aligned sequences were trimmed using trimAlvl.2. Suspected recombinant sequences were identified by running the RDP4 program, running the analysis under seven different methods (RDP, GENECONV, Bootscan, Maxchi, Chimaera, SiSscan, and 3Seq). Modelfinder program was used to carry out a model selection analysis (http://www.iqtree.org/Mode1Finder/). A maximum likelihood phylogeny ofthe dataset was reconstructed by using RAxMLv8.2.12. Maximum-clade credibility trees were obtained using TreeAnnotator (https://beast.community/treeannotator), with 15% burnin, and median heights. We assessed the clocklikeliness of our data by using TempEst vl.5.3 (http://tree.bio.ed.ac.uk/software/tempest/). Phylogeography was reconstructed by using continuous traits (latitudinal and longitudinal coordinates for each sequence) in BEASTvl.10.4 (https://beast.community/). Convergence was assessed using Tracer Vl.7.1 (https://beast.community/tracer). Spatio-temporal patterns of WNV evolution were visualised using SpreaD3 (https://beast.community/spread3).

For manuscripts utilizing custom algorithms or software that are central to the research but not yet described in published literature, software must be made available to editors and reviewers. We strongly encourage code deposition in a community repository (e.g. GitHub). See the Nature Portfolio guidelines for submitting code & software for further information.

Data

Policy information about availability of data

All manuscripts must include a data availability statement. This statement should provide the following information, where applicable:

- Accession codes, unique identifiers, or web links for publicly available datasets
- A description of any restrictions on data availability
- For clinical datasets or third party data, please ensure that the statement adheres to our policy

AII data that support the findings of the study are available from the materiai associateci with Figshare (Project no 160822). Particularly, the accession numbers and curateci metadata of IZS-Teramo and IPD-Dakar West Nile virus genome sequences (Supplementary Table 1), the curated metadata of West Nile virus genome sequences downloaded from NCBI (Supplementary Table 2), the geographic coordinates for sequences analysed in the phylogeographic analysis (Supplementary Table 3), the results of RDP4 for the recombinant sequences (Supplementary Table 4), the root-to-tip divergence analysis table (Supplementary Table 5), and the model selection results (Supplementary Table 6) can be found at dx.doi.org/10.6084/m9.figshare.23660139. Supplementary figures, including the maximum likelihood phylogeny of WNV L1and L2 (Supplementary Figures 1 and 2, respectively), the down sampling of WNV L1 and L2 datasets (Supplementary Figures 3 and 4, respectively), the root-to-tip divergence analysis WNV L1 and L2 (Supplementary Figures 5 and 6, respectively), the sensitivity analysis of WNV L1 and L2 datasets - effect on ancestral node locations (Supplementary Figure 7); and the sensitivity analysis of WNV L1 and L2 - effects on phylogeographic patterns (Supplementary Figures 8 and 9, respectively) can be found at dx.doi.org/10.6084/m9.figshare.23660163. Furthermore, alignments used for the phylogenetic analysis, model selection analysis, and tree files can be found at dx.doi.org/10.6084/m9.figshare.22182418. Finally, phylogeographic inference tree files, geographic coordinates, and videos can be found at dx.doi.org/10.6084/m9.figshare.23660571. The data generated in this study are provided in the Supplementary Information file.

Human research participants

Policy information about studies involving human research participants and Sex and Gender in Research.

Reporting on sex and gender	not applicable
Population characteristics	not applicable
Recruitment	not applicable
Ethics oversight	not applicable

Note that full information on the approval of the study protocol must also be provided in the manuscript.

Field-specific reporting

Please select the one below that is the best fit for your research. If you are not sure, read the appropriate sections before making your selection.

☐ Life sciences ☐ Behavioural & social sciences ☒ Ecological, evolutionary & environmental sciences

For a reference copy of the document with all sections, see nature.com/documents/nr-reporting-summary-flat.pdf

Ecological, evolutionary & environmental sciences study design

All studies must disclose on these points even when the disclosure is negative.

Study description	In this study we used genome sequences obtained from the National Surveillance plan in Italy and Senegal other than public available sequences downloaded from NCBI. The sequence dataset included in this study was used to perform phylogenetic and phylogeographic analyses aimed at uncovering the viral dynamics of West Nile virus between Africa and Europe.
Research sample	Sequences were obtained from samples collected in In Italy between 2001 and 2022 as part of the National Surveillance plan coordinated by the Ministry of Health, the Istituto Superiore di Sanità (epidemiology and national reference laboratory, human), and the Istituto Zooprofilattico of Abruzzo and Molise (IZS-Teramo) (epidemiology and national reference laboratory, animal/entomology) (https://westnile.izs.it/j6_wnd/home, https://www.epicentro.iss.it/westnile/) and in Senegal, where sample activities were carried out between 1988 and 2022 by the Institut Pasteur de Dakar (IPD-Dakar) in collaboration with the Ministry of Health, within the framework of the mosquito-based arbovirus surveillance system and a sentinel syndromic surveillance network (4S).<br>All samples collected in Italy and Senegal were screened by real time PCR for West Nile virus (WNV). Positive samples were sequenced and all newly obtained complete genome sequences, representing the viral WNV population in Italy and Senegal, were considered in this study. Moreover, we retrieved all the WNV available genomes from the NCBI genbank database. All the data and their source are accurately described in Supplementary Tables 1 and 2, as reported in the "Data Availability" statement.
Sampling strategy	The samples used in this study were previously collected in Italy as part of the National Surveillance plan coordinated by the Ministry of Health, the Istituto Superiore di Sanità (epidemiology and national reference laboratory, human), and the Istituto Zooprofilattico of Abruzzo and Molise (IZS-Teramo) (epidemiology and national reference laboratory, animal/entomology) (https://westnile.izs.it/j6_wnd/home, https://www.epicentro.iss.it/westnile/), and in Senegal by the Institut Pasteur de Dakar (IPD-Dakar) in collaboration with the Ministry of Heal within the framework of the mosquito-based arbovirus surveillance system and a sentinel syndromic surveillance network (4S).<br>The samples chosen for this study were all newly generated WNV genome sequences obtained at IZS-Teramo and IPD-Dakar plus all public avaiable WNV NCBI genome sequences.<br>As the minimum sample size for performing tree inference is 4 sequences, and the size of our samples was always greater than 150, the number of samples was more than sufficient to perform a robust phylogenetic and phylogeographic analysis.
Data collection	In Italy, the cases reported in this study were investigated with routine procedures according to the national surveillance plan for arbovirus infection (2020-2025). The integrated surveillance plan is coordinated by the Ministry of Health, the Istituto Superiore di Sanità (epidemiology and national reference laboratory, human), and the Istituto Zooprofilattico of Abruzzo and Molise (IZS-Teramo) (epidemiology and national reference laboratory, animal/entomology) (https://westnile.izs.it/j6_wnd/home, https://www.epicentro.iss.it/westnile/), which perform a coordinated mosquito, human, and animal surveillance.<br>Any positive results from local animal surveillance activities are confirmed by the National Reference Centre for Foreign Animal Diseases (CESME) at IZS-Teramo. Notifications of outbreaks are registered by the official veterinary authorities in the online national information system for the notification of outbreaks in animals (SIMAN) [Colangeli, Patrizia, et al. "The national information system for the notification of animal diseases in Italy." Veterinaria italiana 47.3 (2011): 303-312]. The notification system is designed to register and document the occurrence and evolution of important infectious animal diseases according to Council Directive 82/894/EC. Any human positive cases are collected by the Istituto Superiore di Sanità and reported in specific online platforms of the Ministry of Health.<br>In Senegal, an human surveillance system, coupled with a mosquito-based arbovirus surveillance system, has been set up by the Institut Pasteur de Dakar in collaboration with the Ministry of Health, carrying out a monthly screening of arbovirus and hemorhagic fever virus samples. All data are recorded on scientific laboratory notebooks and computer databases by the WHO collaborating center for arboviruses and hemorhagic fever viruses (CRORA), and reported in specific platforms of the Ministry of Health.
Timing and spatial scale	All samples were collected in Italy between 2008 and 2022 under the National Surveillance plan. Each year, seasonal surveillance activities are defined on the basis of the previous year's virus circulation (risk areas), while any WNV detection in birds, mosquitoes, equids, and humans triggers the activation of prevention measures (blood and transplant measures including nucleic acid testing, vector control, and risk communication campaigns aimed at citizens). Therefore, the start date of these measures can vary each year and, in each province, depending on the viral circulation. Particularly, the surveillance plan includes: i) active surveillance which is made by the fortnightly capture of mosquitoes using a network of fixed traps and the capture of target bird species, and ii) the passive surveillance characterised by the collection of dead-found birds, and by testing cases of neurological disease in horses and viral meningoencephalitis cases in humans. Generally, the active surveillance is performed in high risk regions every year, from the end of April to the beginning of November.<br>In Senegal the samples were collected between 1989 and 2022 by the Institut Pasteur de Dakar (IPD-Dakar), from June to December each year, in the Barkédji area (15°17′ N, 14°52′ W), located in the Sahelian biogeographic domain.
Data exclusions	The L1 sequence OP846974.1 and the L2 sequence OK239667.1 were excluded from our phylogenetic analysis, because detected as recombinant
Reproducibility	The study is fully reproducible by downloading all data from the data repository described in the "Data Availability" statement and by repeating the analyses using the scripts provided in the "Code Availability" statement of our paper.
Randomization	Randomization is often used in clinical trials while here is not applicable because all genome sequences available on the NCBI database plus the ones obtained from WNV positive samples in Italy and Senegal by the Istituto Zooprofilattico of Abruzzo and Molise and the Institut Pasteur de Dakar were included in the phylogenetic and phylogeographic analyses.
Blinding	Blinding was not relevant to this study. Our study includes a phylogenetic and phylogeographic analysis and blinding would have no effect on that. In fact, all genome sequences available on the NCBI database plus the ones obtained from WNV positive samples in Italy and Senegal by the Istituto Zooprofilattico of Abruzzo and Molise and the Institut Pasteur de Dakar were included in the final

dataset and used to perform phylogenetic and phylogeographic analyses.

Did the study involve field work? ☐ Yes ☒ No

Reporting for specific materials, systems and methods

We require information from authors about some types of materials, experimental systems and methods used in many studies. Here, indicate whether each material, system or method listed is relevant to your study. If you are not sure if a list item applies to your research, read the appropriate section before selecting a response.

Materials & experimental systems

n/a	Involved in the study
☒	☐ Antibodies
☐	☒ Eukaryotic cell lines
☒	☐ Palaeontology and archaeology
☐	☒ Animals and other organisms
☒	☐ Clinical data
☒	☐ Dual use research of concern

Methods

n/a	Involved in the study
☒	☐ ChIP-seq
☒	☐ Flow cytometry
☒	☐ MRI-based neuroimaging

Eukaryotic cell lines

Policy information about cell lines and Sex and Gender in Research

Cell line source(s)	Aedes albopictus clone C6/36.
Authentication	C6/36: ATCC number C6/36 CRL-1660TM, Lot number 63675525.
Mycoplasma contamination	Absent
Commonly misidentified lines (See ICLAC register)	None

Animals and other research organisms

Policy information about studies involving animals; ARRIVE guidelines recommended for reporting animal research, and Sex and Gender in Research

Laboratory animals	The study did not involve laboratory animals. It involved the use of Monkey Vero cell lines, Institut Pasteur Paris, Lot number 1/99.
Wild animals	Our study did not involve directly wild animals.
Reporting on sex	Our study did not involve directly wild animals. For this reason this information has not been included in the study
Field-collected samples	Our study did not involve field work. The WNV positive samples we studied were obtained from wild animal samples (mosquito homogenates and wild bird tissue samples) collected under the National Surveillance plan for arbovirus infections. Direct RNA extraction or RNA extraction from tissue cultures (C6/36 and Vero cell lines) were performed in the laboratory.
Ethics oversight	The cases reported in this study were investigated with routine procedures according to the National Surveillance plan for arbovirus infections. Therefore, no approval was required from the ethics committee. No human samples were used in this study.

Note that full information on the approval of the study protocol must also be provided in the manuscript.
